# Mechanistic insights from resolving ligand-dependent kinetics of conformational changes at ATP-gated P2X1R ion channels

**DOI:** 10.1038/srep32918

**Published:** 2016-09-12

**Authors:** Alistair G. Fryatt, Sudad Dayl, Paul M. Cullis, Ralf Schmid, Richard J. Evans

**Affiliations:** 1 Department of Molecular and Cell Biology, University of Leicester, Leicester, LE1 7RH, United Kingdom; 2 Department of Chemistry, College of Science, University of Baghdad, Baghdad, Iraq; 3 Department of Chemistry, University of Leicester, Leicester, LE1 7RH, United Kingdom; 4 Leicester Institute of Structural and Chemical Biology, University of Leicester, Leicester, LE1 7RH, United Kingdom

## Abstract

Structural studies of P2X receptors show a novel U shaped ATP orientation following binding. We used voltage clamp fluorometry (VCF) and molecular dynamics (MD) simulations to investigate agonist action. For VCF the P2X1 receptor (P2X1R) K190C mutant (adjacent to the agonist binding pocket) was labelled with the fluorophore MTS-TAMRA and changes in fluorescence on agonist treatment provided a real time measure of conformational changes. Studies with heteromeric channels incorporating a key lysine mutation (K68A) in the ATP binding site demonstrate that normally three molecules of ATP activate the receptor. The time-course of VCF responses to ATP, 2′-deoxy ATP, 3′-deoxy ATP, Ap5A and αβmeATP were agonist dependent. Comparing the properties of the deoxy forms of ATP demonstrated the importance of the 2′ hydroxyl group on the ribose ring in determining agonist efficacy consistent with MD simulations showing that it forms a hydrogen bond with the γ-phosphate oxygen stabilizing the U-shaped conformation. Comparison of the recovery of fluorescence on agonist washout, with channel activation to a second agonist application for the partial agonists Ap5A and αβmeATP, showed a complex relationship between conformational change and desensitization. These results highlight that different agonists induce distinct conformational changes, kinetics and recovery from desensitization at P2X1Rs.

Extracellular ATP levels can be raised in a variety of ways including release from neurons, through pannexin hemichannels, and due to cell damage^1^,^2^. ATP is an agonist at cell surface P2X receptor (P2XR) channels resulting in membrane depolarization and calcium influx^1^,^2^,^3^. The seven mammalian P2XR subunits (P2X1–7) show a widespread tissue distribution^2^, form homo- and heteromeric receptors with a range of phenotypes^4^ and have important physiological functions^2^,^5^,^6^. This has led to therapeutic interest with subtype selective antagonists showing efficacy as anti-thrombotics (P2X1)^7^, analgesics (P2X3,2/3 &7 refs 8,9) and anti-inflammatory agents (P2X7 ref. 10).

P2XRs comprise a distinct family of ligand gated ion channels^1^,^2^. The crystal structures of the zebrafish P2X4 and gulf coast tick P2XRs show the trimeric assembly of “dolphin” shaped subunits^11^,^12^,^13^ with three ATP molecules bound^12^. However, studies using concatenated receptors suggested that binding of two molecules of ATP can open the channel^14^,^15^. The premise for these studies, that incorporating a mutant subunit into a trimer renders that subunit binding site inactive, has been questioned by work showing agonist binding to one site changes the properties of the remaining sites^16^. Therefore whether the channel normally opens from a bi- or tri-liganded state is unclear.

The P2XR agonist binding site is formed predominantly from conserved residues in the upper and lower body of adjacent subunits^12^. Upon binding, ATP adopts an unusual U shaped conformation that involves rearrangement of the P2XR dorsal fin, left flipper and cysteine rich head region closing the agonist pocket. This is coupled through the lower body to the transmembrane fluke and opening the channel gate. Whether the novel U shape of ATP results from an intramolecular hydrogen bond that contributes to agonist induced conformational changes and/or is driven solely by interactions with the receptor remains to be determined. ATP is not the only natural agonist, diadenosine pentaphosphate (Ap5A), is stored and released from platelets^17^ and acting at P2X1Rs mediates arterial contraction^18^,^19^. The ATP analogue α,β-methylene ATP (αβmeATP) also has agonist activity at P2X1Rs. Interestingly both Ap5A and αβmeATP are partial agonists at P2X1Rs^20^. However, it is unclear whether partial agonist bound receptors show similar conformational transitions as those when ATP is the agonist.

Molecular modelling can simulate drug receptor interactions and help to understand the effects of mutations. Insight into conformational changes can be gained by using voltage clamp fluorometry (VCF). This involves labelling the P2XR^21^,^22^ with cysteine reactive environmentally sensitive fluorophores^23^,^24^. Changes in fluorescence report in real time conformational rearrangements, including those that are electrically silent and cannot be measured by conventional electrophysiological techniques^21^. In this study we have combined molecular modelling and VCF to address (i) how many molecules of ATP are normally required to open P2X1Rs, (ii) the importance of intramolecular hydrogen bonds for ATP agonist action and (iii) conformational changes associated with partial agonist action. Our work provides new insight into fundamental agonist dependent P2XR molecular transitions.

## Results

### How many ATP molecules normally open the P2X1R?

**Figure 1:**
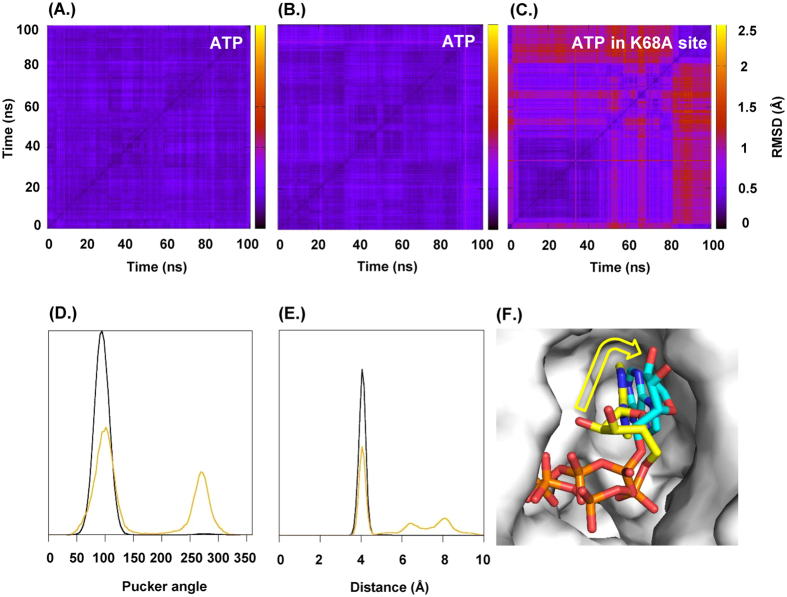
MD simulations of ATP binding at WT subunits and one incorporating the K68A binding site mutation. (**A–C**) 2-D rmsd plots of ATP in P2X1R wild type and P2X1R (K68A) binding sites. 1000 Snapshots were extracted from the 100 ns molecular dynamics trajectories (one snapshot every 10 ps) and used to calculate all-against-all rmsd values for ATP. The rmsd value (in Å) for each calculation is indicated by the colour gradient. ATP bound in a wild type P2X1R binding site (left and centre panel) shows very little variation. In contrast ATP bound to a P2X1R K68A binding site (right panel) shows generally more conformational variability. In the trajectory visualised a conformational transition appears after around 80 ns. (**D**) Ribose pucker phase angle frequency for ATP in P2X1R wild type and K68A binding sites. ATP in the wild type site (black) is almost exclusively in the C2′ endo conformation while in the K68A binding site (orange) C′3 endo conformations are also present (peak at ~270 degree). Values are reported according to the Cremer convention. (**E**) Distance between the gamma phosphate phosphor atom and the C′2 of the ribose. Plotted are the relative frequencies of γ-P – C′2 distances for ATP in a wild type P2X1R binding site (black) and ATP bound to a P2X1R K68A binding site (orange). ATP in the wild type binding site remains in the U shaped conformation, while in K68A binding site additional conformations are accessible indicated by the peaks at ~6.5 Å and ~8 Å. (**F**) Binding conformations for ATP are shown for two snapshots before and after the conformational transition in an overlay corresponding to trajectories at 0.8 ns and 6.8 ns in the right panel of (**C**).

The crystal structures of ATP bound P2XRs show the key role of a conserved lysine (K68, P2X1R numbering) that interacts with all three phosphate groups. Mutant P2X2R K69A (equivalent to P2X1R K68A) homomers were not activated by ATP supporting a critical role of the residue^25^. When the K69A mutant was introduced into one subunit of concatenated trimeric P2X2Rs the channel was still opened by ATP and it was concluded that 2 molecules of ATP can activate the channel^14^. However, it was subsequently shown that binding of the first agonist molecule can change the agonist sensitivity of the remaining binding sites^16^. Therefore, it is unclear whether mutation at a single subunit in a concatenated channel would abolish ATP binding at that site. To probe how ATP binding might be affected molecular dynamics (MD) simulations were performed over 100 ns for the P2X1R containing two wild type subunits and one bearing the K68A mutation. All simulations were stable during the 100 ns time course (Cα-rmsd 2.7 ± 0.2  Å, 2.9 ± 0.3 Å and 2.9 ± 0.2 Å, respectively, see Supplementary Figures 1 and 2). In the non-mutated binding sites the ATP conformation hardly changed from the conformation found in the zfP2X4R X-ray structure (Fig. 1A,B) and the hydrogen bonding pattern (Table 1) is preserved during the simulations. The MD simulations show that ATP at the mutant subunit interface remained within the binding site during the simulation rather than dissociating from the binding site. However the ATP was less constrained in this binding site (Fig. 1C) and sampled additional conformations as indicated by a second peak for the ribose pucker phase angle (Fig. 1D). Also, the presence of key hydrogen bonds was consistently lower (Table 1). In particular the intramolecular hydrogen bond between the 2′-hydroxy group of the ribose and the γ-phosphate was only present in less than 50% of the frames (Fig. 1E). Within the timeframe of the simulations no effect of the K68A substitution on the non-mutated binding sites was observed. These MD simulations suggest that ATP may still be able to bind at all three sites of a P2XR incorporating one mutated binding site, however with more conformational variation at the mutated binding site (e.g. Fig. 1F). This questions the assumption that the K68A site cannot bind ATP at all, and hence the interpretation of experiments incorporating binding site mutant subunits in concatenated channels.

**Figure 2:**
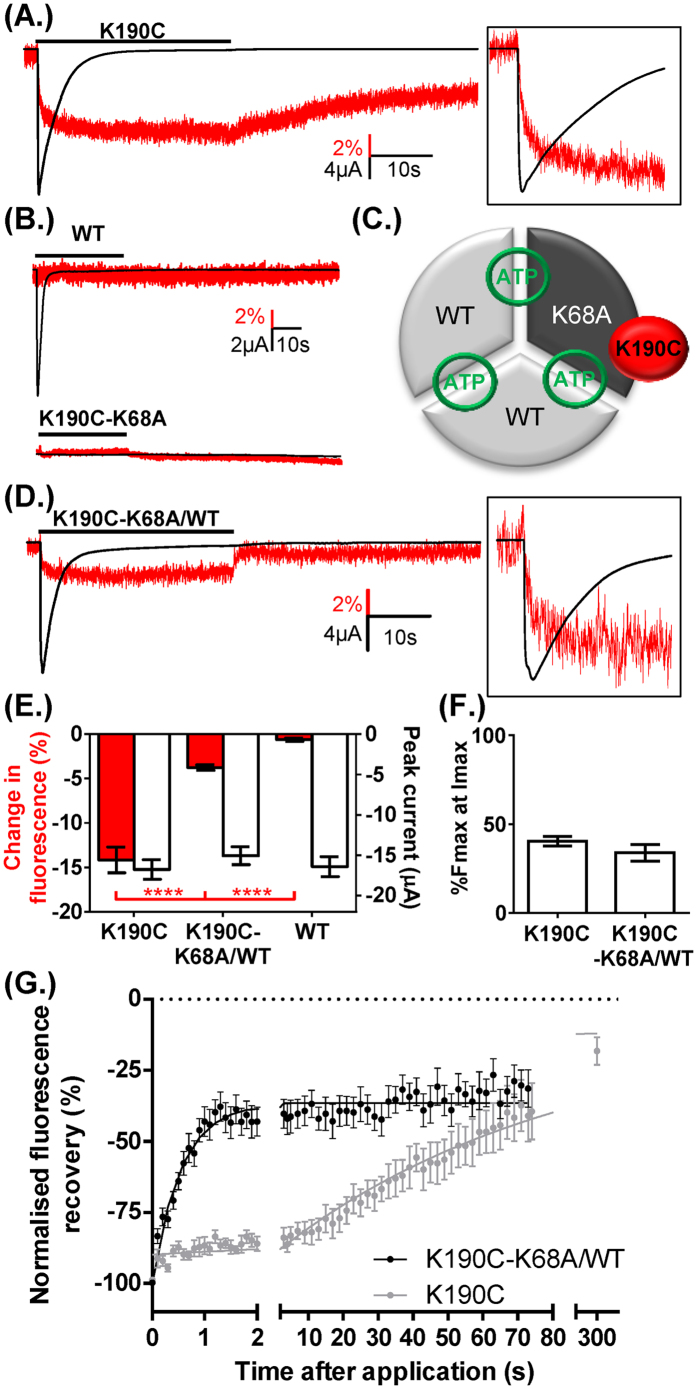
Fluorescence changes can be rescued in ATP insensitive subunits by co-expression of WT subunits. (**A**) Example VCF traces from K190C showing rapidly desensitizing current and decrease in fluorescence with ATP (100 μM). Insert shows expanded timescale during initial ATP application, with fluorescence change normalized to peak current. (**B**) Example of VCF traces from oocytes expressing wild type hP2X1R (upper panel) and double mutant P2X1R K190C-K68A (lower panel). Both show no change on fluorescence with 100 μM ATP application (30 s indicated by bar) and no current was evoked from K190C-K68A. (**C**) Schematic showing a heteromeric receptor with two WT and one K190C-K68A mutant subunit (grey), inter-subunit binding sites are shown in green and labelling of the mutant subunit by cysteine reactive MTS-TAMRA (in red). (**D**) The ATP evoked current and fluorescence changes were recovered by co-expressing K190C-K68A with WT P2X1R, although the fluorescence change was significantly smaller than those seen for K190C. Insert shows expanded timescale during initial ATP application, with fluorescence change normalized to peak current. (**E**). There was no difference in the %Fmax at Imax between K190C and K190C-K68A/WT (**F**). (**G**) The recovery of the fluorescence change following agonist wash out was significantly faster for K190C-K68A/WT compared to K190C (traces normalized to peak decrease in fluorescence), with time from 90% to 50% fluorescence recovery of 0.7 ± 0.3 s and 38.8 ± 4.7 s respectively. Data are shown as mean ± SEM, n ≥ 6, ****p < 0.0001.

To test the effects of a mutant binding site on P2X1R properties we have used VCF to measure conformational changes at receptors incorporating a fluorescently labelled subunit with a mutated ATP binding site. Attempts to make functional P2X1R concatenated channels have been unsuccessful (ref. 26 and unpublished observations). Therefore, we have used co-expression of WT and mutant P2X1Rs. This results in a mixture of channel combinations that can make interpretation of results difficult. However we have been able to simplify the analysis by using a VCF based approach to measure changes at only the heteromeric channels. The P2X1R mutant K190C (close to the ATP binding pocket) can be labelled with cysteine reactive fluorescent MTS-TAMRA^21^. Following labelling, ATP (100 μM) evoked transient inward currents that desensitized during continued agonist application. ATP decreased MTS-TAMRA fluorescence with two phases; a fast initial phase time-locked with the rise in inward current (~45% maximum fluorescence decrease at peak inward current) and a secondary slower phase corresponding to the channel entering the desensitized state. On ATP washout the fluorescence returned to resting levels (Fig. 2A). MTS-TAMRA does not label WT P2X1Rs^21^, and in control studies ATP application had no effect on fluorescence levels at oocytes expressing WT P2X1Rs treated with MTS-TAMRA. At the K190C receptor that was mutated to remove the critical lysine residue (K190C-K68A) ATP had no effect on membrane current or fluorescence (Fig. 2B). These results are consistent with our previous study and show that VCF can be used to measure the time-course of conformational transitions in the P2X1R associated with activation, desensitization and recovery on agonist washout^21^.

The ATP binding site is formed at the subunit interface^12^,^27^ with K68 and K190 on the same right hand subunit of the binding pocket. Co-expressing WT and K68A-K190C mutants is expected to give rise to homomeric and heteromeric subunit combinations. As no fluorescence change was recorded from homomeric WT or K190C-K68A receptors (Fig. 2B) any changes in MTS-TAMRA fluorescence following co-expression will provide a direct measure of conformational changes at heteromeric channels incorporating the K68A mutation (Fig. 2C). We were therefore able to determine whether the VCF profile is similar for receptors with three equivalent ATP binding sites and those incorporating mutant binding site(s). When WT and K68A-K190C P2X1R subunits were co-expressed ATP (100 μM) evoked an ~3% decrease in MTS-TAMRA fluorescence (Fig. 2D,E). This “rescue” of an ATP evoked fluorescence decrease on co-expression of subunits (that show no fluorescence change to ATP when expressed homomerically) demonstrates that heteromers are formed. The concatenated studies on P2X2Rs indicated that incorportation of two mutant subunits essentially abolished channel function^14^. We therefore interpret our fluorescence data as resulting from the incorporation of a single mutant K68A-K190C subunit with two WT subunits to form the heteromer. The time-course of the decrease in MTS-TAMRA fluorescence is equivalent between K190C and K68A-K190C-WT heteromers with an intial component time-locked with the rise of the ATP current (%Fmax at Imax 40.5 ± 2.7% and 34.0 ± 4.6% respectively, 10–90% current rise times of 165.6 ± 29.5 and 114.2 ± 14.9 ms respectively) and a secondary slower component consistent with the channel entering the desensitized state. This suggests that the time course of channel opening and desensitization was unaffected by incorporation of the binding site mutation in the heteromers. Interestingly, the time-course of recovery of the fluorescence on washout of ATP was ~60 fold faster for the heteromer (time to 50% recovery 38.8 ± 4.8 and 0.7 ± 0.4 s for K190C and K68A-K190C/WT heteromer respectively, p < 0.0001)(Fig. 2G). This clearly indicates that the conformational changes in the receptor on agonist washout are determined by the characteristics of the ATP binding sites. If the channel could open normally with less than three molecules of ATP bound the washout rate would have been expected to be the same as for K190C (see discussion). The more rapid recovery in fluorescence of the K190C-K68A heteromer shows that normally three ATP molecules of ATP bind to open the P2X1R.

### The 2’ ribose ring hydroxyl group imparts agonist efficacy

**Figure 3:**
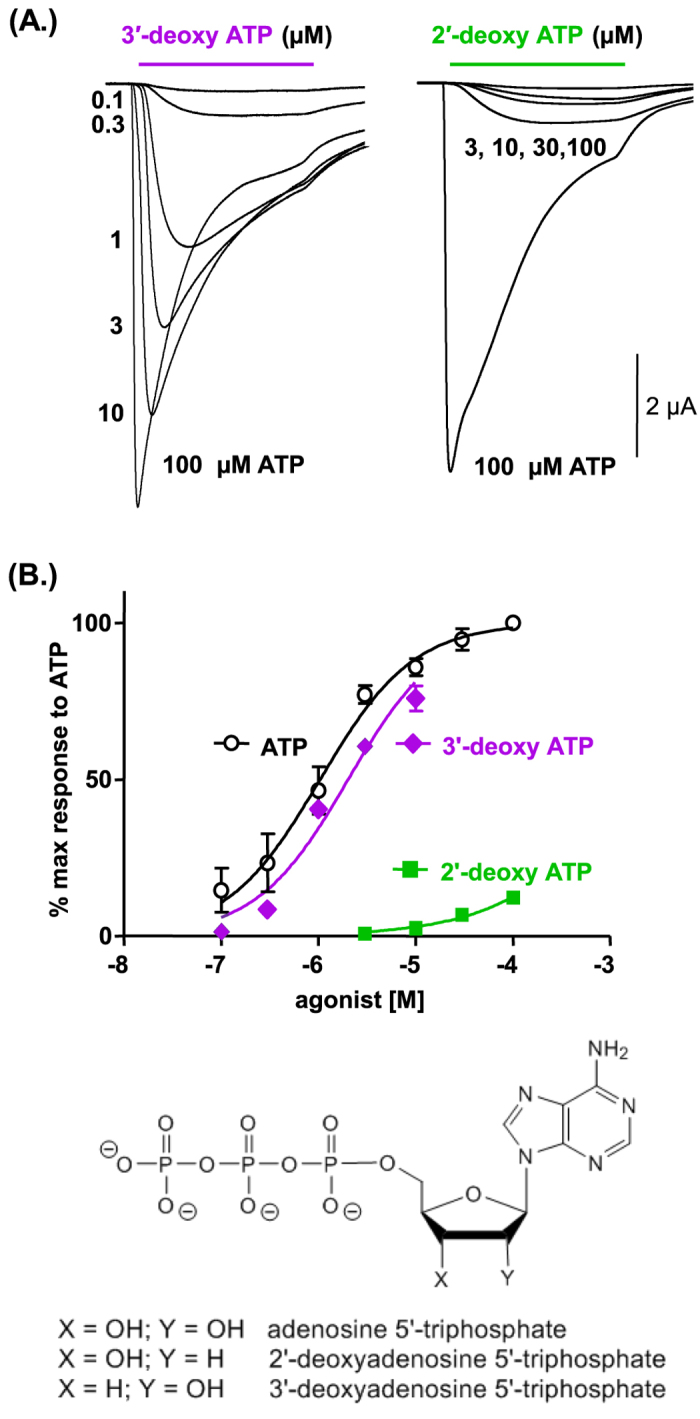
The hydroxyl group at the 2′, but not 3′, position on the ribose ring of ATP is important for agonist action at P2X1Rs. (**A**) Agonist evoked currents (3 s application indicated by bar) at P2X1Rs. Representative traces from a single oocyte are shown for 3′-deoxy ATP and 2′-deoxy ATP (a response to a maximal concentration of ATP 100 μM from the same oocyte is shown for comparison). (**B**) Concentration response curves for agonist evoked currents, ATP (open circles), 3′-deoxy ATP (purple diamonds) and 2′-deoxy ATP (green squares). Due to cost a maximal concentration of 10 μM 3′-deoxy ATP was used. Data are shown as mean ± SEM, n = 4.

The crystal structure of the zfP2X4 receptor^12^ shows bound ATP adopts a U shape with the gamma phosphate and the hydroxyl group at the 2′ position on the ribose in close proximity. This raised the possibility that a hydrogen bond between these groups contributes to the stabilisation of the conformation. To determine the importance of the hydroxyl groups we tested the effect of removing them at either the 2′ position (2′-deoxy ATP) or the 3′ position (3′-deoxy ATP). 3′-deoxy ATP evoked transient inward currents that were essentially the same as those in response to ATP (EC_50_ ~2 and 1 μM for 3′-deoxy ATP and ATP respectively). In contrast 2′-deoxy ATP was ineffective as an agonist (responses to 100 μM were only 12.3 ± 2.1% of the maximum response to ATP, n = 4)(Fig. 3). These results suggest that the hydroxyl group at the 2′ position plays an important role in agonist action at the P2X1R.

**Figure 4:**
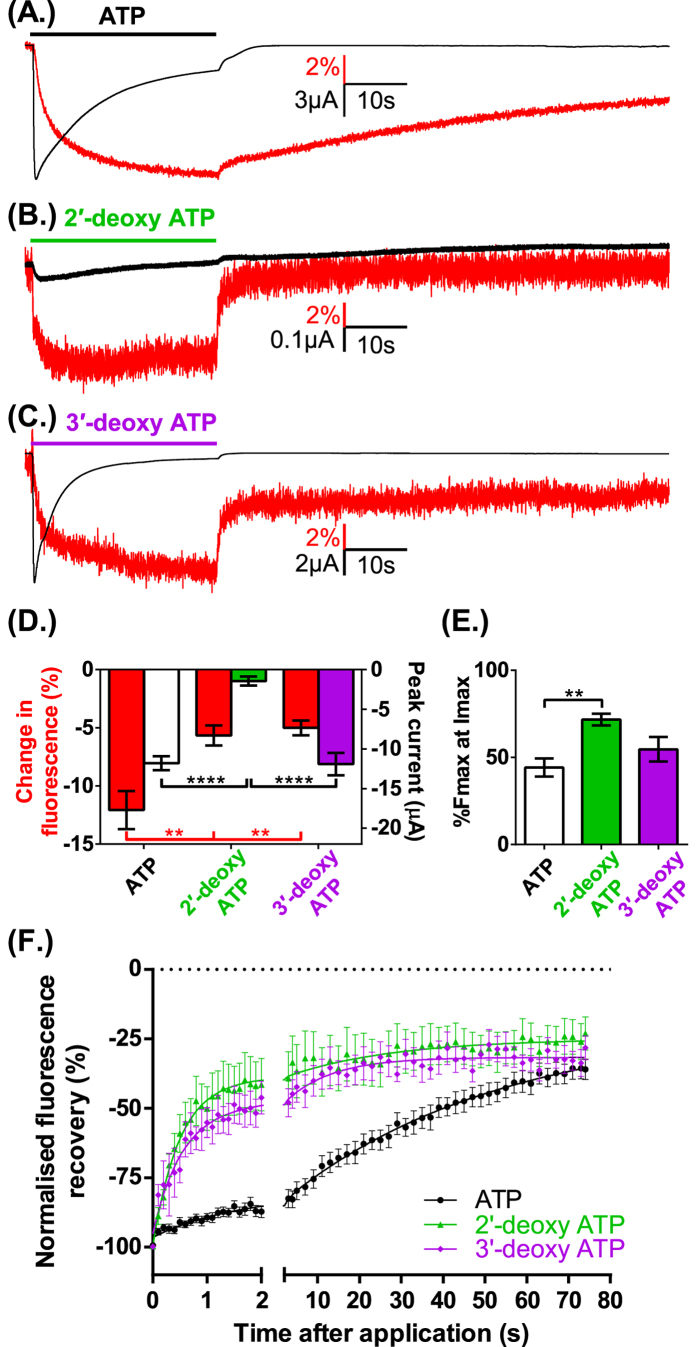
VCF reports changes in fluorescence induced by deoxyadenosine 5′-triphosphates. (**A**) Example of VCF traces from oocytes expressing P2X1R K190C (10 μM ATP indicated by bar, black trace = current recording, red trace = fluorescence). Inward currents and decreases in fluorescence were also seen with the application of (**B**) 2′-deoxy ATP and (**C**) 3′-deoxy ATP (both 10 μM). The fluorescence changes were significantly smaller than those seen with ATP while 2′-deoxy ATP evoked a significantly reduced current (**D**) The proportion of the fluorescence change at peak current (%Fmax at Imax, (**E**)) was also significantly greater that ATP with the application of 2′-deoxyATP. (**F**) The recovery of the fluorescence change following agonist wash out was significantly faster for both 2′-dATP and 3′-deoxy ATP compared to ATP, with time from 90% to 50% fluorescence recovery of 1.0 ± 0.5 s, 2.5 ± 0.9 s and 30.4 ± 2.0s respectively. Data are shown as mean ± SEM, n ≥ 5, **p < 0.01, ****p < 0.0001.

The weak partial agonist activity of 2′-deoxy ATP could result from changes in binding and/or a reduction in channel gating. We therefore used VCF to measure in real time conformational changes in the receptor (that may be electrically silent). 3′-deoxy ATP (10 μM) evoked currents had a similar time-course and peak current amplitude to those evoked by ATP (10 μM). 2′-deoxy ATP (10 μM) evoked currents were only ~5% (p < 0.0001) of the peak ATP response, and had a slower time-course of desensitization (Fig. 4). In contrast to their marked differences in peak current amplitude 2′-deoxy and 3′-deoxy ATP evoked similar amplitude decreases in MTS-TAMRA fluorescence (~ 5%)(Fig. 4). The ~70% decrease in fluorescence at the peak current for 2′-deoxy ATP (%Fmax at Imax) suggests that this change in fluorescence is dominated by the initial agonist binding step. For 3′-deoxy ATP the decrease in fluorescence reflects ~55% associated with the initial agonist binding step and the residual ~45% with channel desensitization (Fig. 4). The equivalent peak decrease in fluorescence for the deoxy analogues indicates that they both bind to the receptor and that the reduced current/partial agonist activity of the 2′-deoxy form results predominantly from decreased gating to the open state. The 2′-deoxy or 3′-deoxy ATP evoked fluorescence changes returned to baseline following agonist washout with initial rapid (50% within 2 s) and secondary slower components (~ 75% recovery at 75 seconds washout). The initial recovery was considerably faster than for ATP and shows that deoxy ATP analogues have different binding characteristics/conformations than ATP (Fig. 4). The hydroxyl groups on the ribose are recognized by the dorsal fin in the crystal structure of the zfP2X4R^12^,^28^. The faster recovery on agonist washout for the deoxy forms suggests that interactions of both the hydroxyl groups contribute to the kinetics of recovery from the ATP bound conformation.

### MD simulations highlight the importance of the 2’ hydroxyl group for the “U” shaped ATP binding conformation

**Figure 5:**
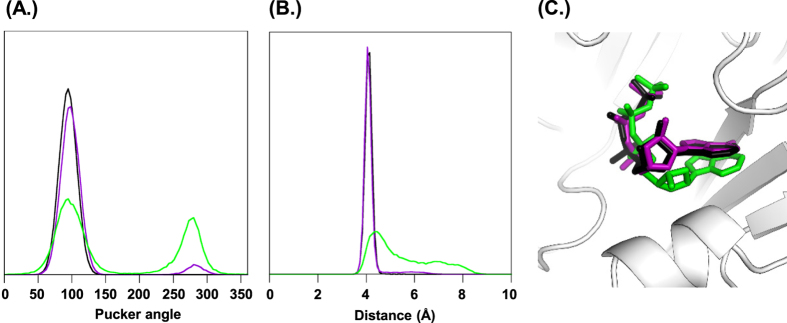
Conformations of ATP, 3′-deoxy ATP and 2′-deoxy ATP in the P2X1R binding site. (**A**,**B**) Nucleotide conformations in the MD simulations extracted from a total of six traces (three binding sites, two replicates) are monitored via their ribose pucker phase angle (**A**) and the distance between the gamma phosphate phosphor atom and the C′2 of the ribose (**B**) Plotted are the relative frequencies of the respective angle (3.6 degrees window) and distance (0.1 Å window) for ATP (black), 3′deoxy-ATP (purple) and 2′-deoxy-ATP (green). The second peak in the ribose pucker phase angle distribution and the broad shoulder in the distribution of 2′-deoxy ATP γ–phosphate C′2-ribose distances indicates at least two conformational states. (**C**) Overlay of ATP (black), 3′-deoxy-ATP (purple), and the non U shaped 2′-deoxy ATP (green) conformation, the P2X1R is shown as a cartoon, the nucleotides are shown as stick models.

In the context of their different agonist effects on the P2X1R the conformational variation of ATP, 2′-deoxy ATP and 3′-deoxy ATP bound to the P2X1R was investigated via a set of comparative MD simulations. In the starting structures ATP and its derivatives were bound in the conformation as found for ATP in the zfP2X4R X-ray structure. All production simulations were stable within the 100 ns time course with Cα-rmsd between 2.6 and 2.9 Å(Supplementary Figure 1) and the three compounds remained bound to the receptor over the full length of the simulations. The MD trajectories were then analysed for variation in the conformations of bound ATP, 2′-deoxy ATP and 3′-deoxy ATP. ATP and 3′-deoxy ATP show very little variability with an all against all rmsd (based on snapshots taken every 10 ps) of 0.47 ± 0.08 and 0.48 ± 0.14 Å respectively. In contrast, the all against all rmsd of 0.75 ± 0.23 Å in the 2′-deoxy ATP simulations indicates more conformational variation. The experimentally determined ATP binding mode in zfP2X4R is characterised by a series of hydrogen bonds between ATP and highly conserved residues of the P2XR^12^. For instance, the ATP phosphate groups were directly coordinated by the charged and polar side chains of K68, K70, N290, R292 and K309 (residue numbering refers to P2X1). While all these interactions were present in the ATP, 3′-deoxy ATP and 2′-deoxy ATP simulations, they tend to be less persistent in the case of the 2′-deoxy ATP simulations consistent with the greater variability seen in 2′-deoxy ATP conformations (Table 1). The binding of the adenine moiety was characterised by hydrogen bonds between the adenine amine and backbone carbonyl groups of T186 and K68, and a hydrogen bond between the hydroxyl group of the T186 side-chain and the aromatic ring nitrogen N1 of the adenine group. For all three compounds the adenine group remains within the binding cavity during the time course of the simulations and the hydrogen bonding pattern between the adenine moieties of ATP, 3′-deoxy ATP and 2′-deoxy ATP, and the P2X1R was remarkably similar. The simulations show little difference in the distances between the side chain OH-oxygen of T186 and the N1 of the adenine moieties (3.1 ± 0.4 Å, 3.2 ± 0.4 Å and 3.3 ± 0.4 Å for ATP, 3′-deoxy ATP and 2′-deoxy ATP, respectively), between the carbonyl oxygen of T186 and the adenine amine-nitrogen (2.9 ± 0.2 Å, 3.0 ± 0.2 Å and 3.3 ± 0.3 Å), and between the carbonyl oxygen of K68 and the adenine amine-nitrogen (3.0 ± 0.1 Å, 3.0 ± 0.2 Å and 3.6 ± 0.4 Å). There were however remarkable differences in the intramolecular hydrogen bonding patterns of ATP, 3′-deoxy ATP and 2′-deoxy ATP. Intramolecular hydrogen bonds between γ-phosphate oxygen and ribose hydroxyl groups were present in 98 ± 1% of the ATP simulation snapshots and 91 ± 6% for 3′-deoxy ATP. During both, ATP and 3′-deoxy ATP simulations such intramolecular hydrogen bonds occur exclusively via the 2′-hydroxyl group of the ribose. As 2′-deoxy ATP lacks the relevant hydroxyl group the equivalent interaction cannot occur. In addition no compensating hydrogen bond involving the 3′-hydroxyl group of the ribose was found. The lack of such intramolecular hydrogen bonds allows for more flexibility of the ribose moiety as measured by the ribose pucker angle (Fig. 5) of 2′-deoxy ATP. This was also reflected in the variation of the distances between the γ-phosphate, and the C2′ and C3′ atoms of the ribose ring (Fig. 5). In essence, these distances are a descriptor of the orientation of the ribose ring relative to the γ-phosphate group, and hence how far a conformation resembles the U-shape of the ATP conformation seen in the zP2X4 X-ray structure. Analysis of the trajectories shows that 2′-deoxy ATP binds within the agonist pocket (consistent with the VCF data) but does not readily form the U shaped conformation seen for ATP and suggests that this underlies its weak partial agonist activity.

### VCF profile of partial agonist ATP analogues Ap5A and αβmeATP

**Figure 6:**
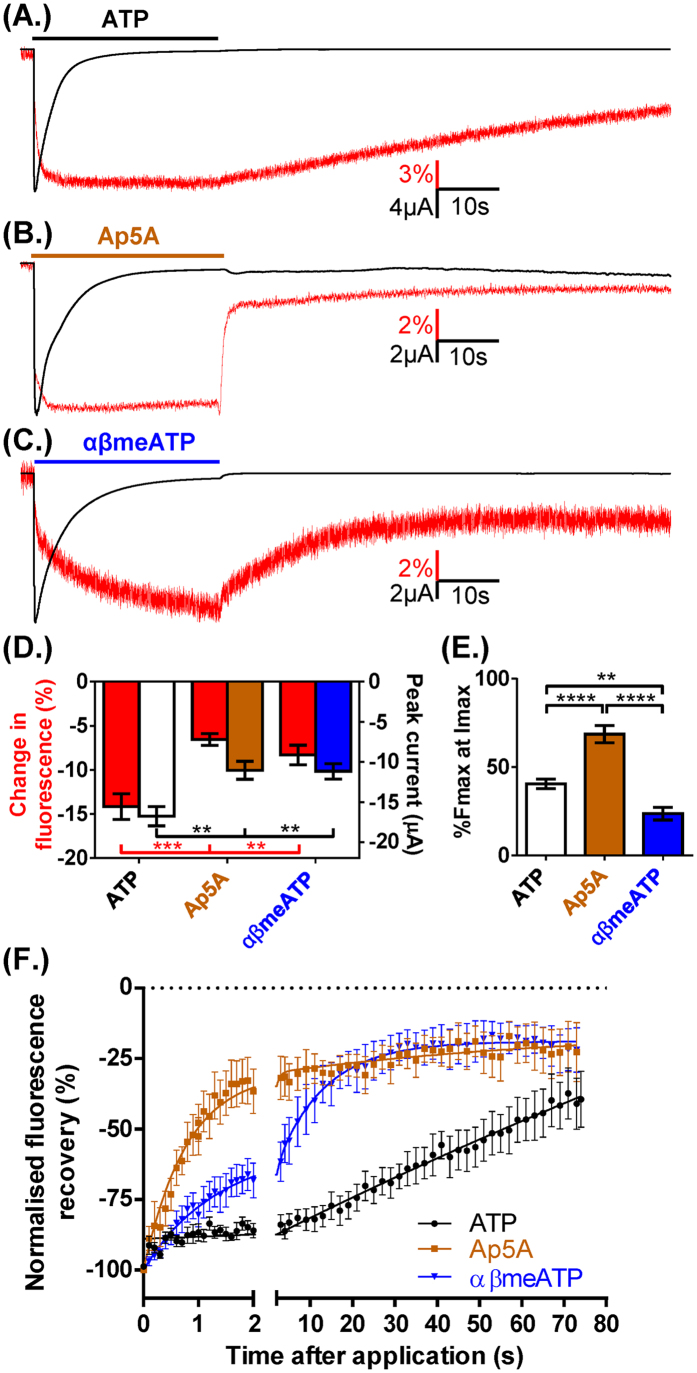
VCF profile is ATP analogue dependent. (**A**) Example of VCF traces from oocytes expressing P2X1R K190C (100 μM ATP indicated by bar). Inward currents and decreases in fluorescence were seen with the application of **(B)** Ap5A and **(C)** αβmeATP (100 μM). The fluorescence changes induced by αβmeATP were significantly smaller than those seen with ATP while both Ap5A and αβmeATP generated significantly reduced currents (**D**). The proportion of the fluorescence change at peak current (%Fmax at Imax, (**E**)) was significantly greater with the application of Ap5A while significantly reduced with αβmeATP application. **(F**) The recovery of the fluorescence change following agonist wash out was significantly faster for both Ap5A and αβmeATP compared to ATP, with time from 90% to 50% fluorescence recovery of 1.4 ± 0.4 s, 6.5 ± 1.4 s and 38.8 ± 4.7 s respectively. Data are shown as mean ± SEM, n ≥ 4, *p < 0.05, **p < 0.01, ****p < 0.0001.

The ATP analogues Ap5A and αβmeATP are partial agonists at the P2X1R^19^,^20^ and we were interested to determine whether they exhibited similar conformational changes to ATP, i.e. VCF profiles. A maximal concentration (100 μM) of either Ap5A or αβmeATP evoked rapidly desensitizing inward currents with essentially the same time course as those to ATP (Fig. 6). The peak current amplitude for Ap5A or αβmeATP currents was ~65% of those to ATP consistent with their partial agonist action (Fig. 6D). The amplitude of the decrease in MTS-TAMRA fluorescence was dependent on the agonist (ATP > αβmeATP ≥ Ap5A). We do not think the reduced fluorescence change for the partial agonists results from reduced binding for two reasons; (i) previous radioligand binding studies have shown that 1 μM αβmeATP essentially abolished radiolabelling with ATPγS at recombinant P2X1Rs^29^ indicating that at the concentration used in this study (100 μM) the P2X1Rs are likely to be saturated and (ii) 3′-deoxy ATP, that was a full agonist, gave a decrease in MTS-TAMRA fluorescence was also less than half that for ATP. The reductions in fluorescence had initial fast components linked to the rise time of the current, and slower secondary decreases linked to desensitization as described previously for ATP evoked responses^21^. However, the relative proportions of the initial and secondary decreases in fluorescence were significantly different between the agonists. For Ap5A ~75% of the fluorescence decrease was associated with the initial agonist application and rise time of the P2X1R current (%Fmax at Imax), this was ~45% for ATP (consistent with previous work)^21^ and was ~30% for αβmeATP.

**Figure 7:**
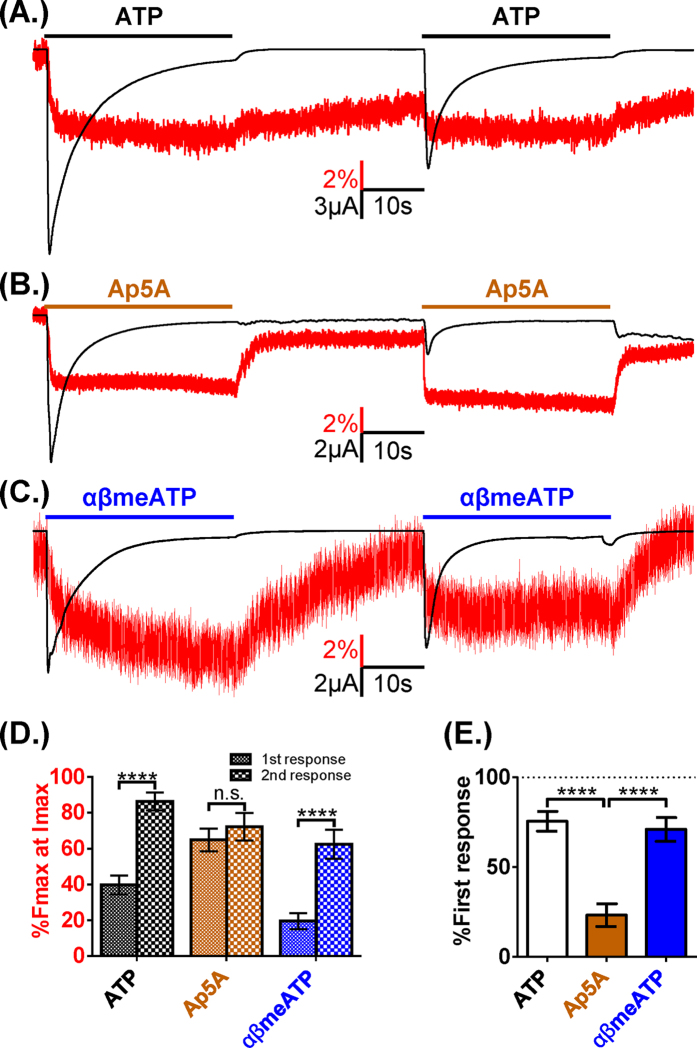
Differential recovery of fluorescence and channel activity from activation by ATP analogues. (**A**) Example of VCF traces from oocytes expressing P2X1R K190C to two 30 second ATP applications (100 μM indicated by bar) with 30 second washout between. A sustained fluorescence decrease was observed during the first agonist application that partially recovered with washout. The subsequent application induced a smaller peak current with a decrease in fluorescence to a similar level as previous. (**B**) Replacing ATP with Ap5A (100 μM) in the above protocol generated an attenuated current during the second application with a similar decrease in fluorescence as the initial application. The response seen with αβmeATP (**C**) was similar to that seen with ATP. Measuring the average %Fmax at Imax during the first and second applications (**D**) showed no change in the proportion of the fluorescence change with application of Ap5A, while both ATP and αβmeATP showed more rapid change in fluorescence with the subsequent agonist trial. (**E**) Graph showing the average evoked current during the second agonist application as a percentage of the first, with Ap5A showing a significantly reduced current compared to ATP or αβmeATP. Data are shown as mean ± SEM, n ≥ 3, ****p < 0.0001.

There were marked differences in the return of fluorescence on agonist washout; Ap5A and αβmeATP had fast and slow components whilst recovery from ATP could be fit with a single slow component. The relative speeds of initial recovery (measured as time of 90 to 50% recovery) were Ap5A >  αβmeATP > ATP (1.4 ± 0.4, 6.5 ± 1.4 and 38.8 ± 4.8 s respectively). Studies on the P2X3R have suggested that recovery from the desensitized state is dependent on the agonist^30^. Given the differences in time-course of recovery for MTS-TAMRA fluorescence on washout between agonists we were therefore interested to see if this was also the case at the P2X1R. Following desensitization the agonist was washed out and then re-applied 30s later. The currents were 75.5 ± 5.5, 23.2 ± 6.3 and 71.0 ± 6.6% of the control response for ATP, Ap5A and αβmeATP respectively (Fig. 7). For all agonists the change in fluorescence signal for the second application was faster than that to the first application (Fig. 7). These results show that the fast initial recovery of fluorescence for Ap5A and αβmeATP is unlikely to reflect the time-course of the channel coming out of the desensitized state. The unique ligand dependent differences in VCF time-course/profile, and recovery from desensitization for Ap5A, αβmeATP and ATP highlight variations in the modes of agonist action at the P2X1R and show recovery from desensitization and changes around the binding pocket are not directly linked.

## Discussion

The crystallization of ATP bound P2XRs provided a 3D picture of the agonist binding site, highlighted the molecular interactions of ATP with several conserved amino acids^12^,^13^, and forms an essential framework for understanding the molecular properties of P2XRs. In this study we have used MD simulations and real time VCF characterization of conformational changes to address important questions about agonist action at P2XRs.

The suggestion that P2XRs could open with less than three molecules of ATP bound^14^,^15^ raised the question of “how many molecules normally bind to the receptor to activate it”? We addressed this by measuring fluorescence changes in heteromeric receptors incorporating a mutation (K68A) at one subunit interface that when expressed as a homomer abolished ATP action. We showed that the time-course of fluorescence change associated with channel activation and desensitization was indistinguishable from the channels with three normal binding sites and this is consistent with studies on P2X2R concatenated channels^14^. However, we were also able to measure the electrically silent conformational changes on ATP washout (that were not addressed in the P2X2 concatenated receptor). The recovery was ~60 fold faster and clearly shows that the mutant binding site contributes to P2X1R properties. In addition our MD simulations predict that ATP can bind to the mutated subunit, albeit with reduced stability. Our work clearly shows that incorporation of the mutant subunit changes the properties of the receptor. This questions the premise of the P2X2R concatenated study that the incorporated mutant binding site would be non-functional, and the conclusion based on it that the P2XR can open with only two molecules of ATP bound. Our VCF studies and MD simulations suggest that ATP binds to the mutant subunit, but does it also contribute to channel gating? Previous single channel studies on the P2X2 receptor showed that the equivalent lysine mutation (K69A) does not interfere with channel gating^31^ (compared to lysine residue 308 that contributes to the ATP binding site and channel gating). We therefore suggest that conformational changes induced by ATP binding to the two normal “high affinity” binding sites facilitate binding at the remaining mutant site and gating of the channel. This is supported by a study showing that binding of the first agonist molecule changes the sensitivity to additional ligand steps; for example this can result in P2X2Rs becoming sensitive to ligands that were previously ineffective e.g. ADP^16^. It therefore seems most likely that the heteromeric channel incorporating a K68A subunit opens with three ATP molecules bound. We propose that the faster initial washout in the heteromeric receptor incorporating the binding mutation results from reduced affinity of the K68A subunit so the ATP is able to dissociate more quickly and this accounts for the more rapid recovery. The fact that when there are three normal ATP binding sites the recovery is slower on washout indicates that channel opening usually results from the binding of three ATP molecules. This is consistent with the finding that the kinetic behaviour of P2XR single channel recordings was fitted best with a model that incorporates three molecules of ATP binding with positive co-operativity^32^. Therefore our studies support the idea that the P2X receptor channel normally opens with three molecules of ATP bound.

Pharmacological studies showed the requirement of the triphosphate tail and adenine ring for agonist action at P2XRs that are consistent with key interactions identified in structural studies^12^. However, the importance of the hydroxyl groups on the ribose ring was unclear. We showed that 2′-deoxy ATP was only poorly effective in opening the channel i.e. it is a very weak partial agonist (we found similar very weak partial agonist activity at the P2X2R – data not shown) whilst 3′-deoxy ATP was a full agonist at the receptor. The reduced efficacy of 2′-deoxy ATP did not result from a reduction in agonist binding as VCF showed that 2′ and 3′-deoxy ATP bind to the receptor at equivalent levels (same amplitude VCF change). This is consistent with early radioligand binding studies on native P2X1Rs showing similar displacement of α,β-methylene ATP binding for 2′-deoxy and 3′-deoxy ATP^33^. Our MD simulations suggest that the U-shaped conformation within the bound ATP is central to high agonist efficacy. Within the 100 ns timeframe of the MD simulations, ATP and 3′-deoxy ATP maintain the U shaped conformation found in the zfP2X4 X-ray structure. However, in the 2′-deoxy ATP simulations conformations different from the U-shape were found within the short timeframe of the simulations. These data suggest that 2′-deoxy ATP might bind to P2X1R in alternative conformations. Consequently, considering the very weak agonist activity of 2′-deoxy ATP this binding is not sufficient for opening of the P2XR ion channel. This also suggests that the U shaped ATP conformation plays a crucial role in structural changes leading to channel opening following agonist binding and is a key determinant of efficacy.

Ap5A and αβmeATP were partial agonists at the P2X1R evoking ~65% of the maximal ATP response, efficacies similar to that reported previously^20^. A recent NMR study on zfP2X4R measuring changes in orientation of methionine residues in the lower body and pore region suggested that the partial agonist activity of αβmeATP results from a gating effect^28^. However, close to the binding site, they were only able to look at a methionine residue on the right flipper where αβmeATP evoked similar changes as ATP^28^. We have been able to detect conformational changes adjacent/just below the agonist binding site. Our work shows that the VCF profiles of ATP, Ap5A and αβmeATP were different indicating marked variations in agonist binding modes, and hence that the differences in conformation around the agonist binding pocket contribute to efficacy. The importance of the U-shaped conformation of ATP in agonist efficacy was suggested from the deoxy ATP studies. For Ap5A the ADP group added to ATP provides “bulk” that may hinder sterically the U shaped conformation and the methylene group between the alpha and beta phosphates could interfere with the flexibility of αβmeATP. It is therefore tempting to speculate that the partial agonist actions of Ap5A and αβmeATP result from a reduced ability to adopt the U-shaped conformation and thus efficacy.

VCF provides a real-time output of the local environment of the introduced MTS-TAMRA. This is particularly useful as it provides a means to study the conformational rearrangements following agonist washout. These fluorescence recovery rates were markedly different between agonists and clearly highlight that different agonists have different conformational signatures. Interestingly the recovery of P2X1R currents from the desensitized state was not directly linked to the recovery of VCF signal for any of the agonists tested. This was most apparent when comparing Ap5A and ATP; where for Ap5A recovery of fluorescence after 30 s wash was more rapid and complete than for ATP and yet there was ~50% less recovery in the current response to a second agonist application. Our results extend our previous work^21^ and show that the rate of current recovery from desensitization is dependent on the agonist and not directly related to the return of fluorescence (i.e. conformation) measured in the extracellular domain close to the ATP binding pocket. Given the rigid coupling of the ATP binding region to the channel gate^12^ one would expect on agonist washout the reversal of the conformational change at the agonist binding site to return the channel gate to the resting conformation that can be then reactivated by agonist binding. However our results clearly show that changes in conformation close to the agonist binding site do not correlate with recovery from desensitization. This suggests that channel opening triggers an additional mechanism that stops ionic permeation i.e. a distinct desensitization gate. The agonist dependent differences in time-course of VCF profiles and current recovery from desensitization clearly indicate a complex relationship between conformational changes around the agonist binding site and the state of the receptor channel that will require further studies to address.

The VCF technique has allowed us to characterize fundamental agonist dependent conformational changes that cannot be addressed by conventional electrophysiological recoding. By measuring electrically silent conformational changes in the P2X1R on agonist washout we have been able to show that the profile is agonist dependent and demonstrate that normally three molecules of ATP bind to activate the receptor. In addition we have shown that the interaction of the 2′ hydroxyl group on the ribose ring with the γ-phosphate oxygen plays a central role in determining agonist efficacy and we suggest partial agonist action results from a reduced ability of the agonist to adopt the U-shaped conformation of ATP seen in the zfP2X4R structure.

## Methods

### Homology modelling and molecular dynamics simulations

Homology models of the trimeric P2X1R and a variation of the receptor comprising two wild-type subunits and one subunit bearing a K68A mutation were built in Modeller 9 v14^34^ based on the zfP2X4 open structure 4DW1^12^ and ranked according to their DOPE score 35. The best ranked models were used as starting structures for a series of comparative molecular dynamics (MD) simulations of the P2X1R extracellular domains with ATP, 2′-deoxy ATP, and 3′-deoxy ATP bound. All MD simulations were performed in Amber14 using the ff14SB and GAFF forcefields^36^. Force field parameters for ATP, 2′-deoxy ATP, and 3′-deoxy ATP were obtained from the AMBER parameter database^37^ or where not available derived from ff14SB and GAFF. RESP charges were calculated in Antechamber based on Gaussian 03 at HF/6–31G* theory level. The starting structures were energy minimized and equilibrated following a protocol described previously^38^. Production simulations were run at 300K for 100 ns in at least two independent replicates on the University of Leicester HPC computing cluster. Molecular dynamics trajectories were analysed within the amber cpptraj module^39^. Due to the trimeric structure of the P2XR three binding sites are simulated within each replicate, hence measurements are reported as mean values and standard deviations of six time series unless stated otherwise.

### Expression of human P2X1Rs in Xenopus laevis oocytes

Manually defolliculated stage V *Xenopus laevis* oocytes were injected with 50 nl (50 ng) of wild type or mutant human P2X1R cRNA using an inject + Matic microinjector (L. A. Gaby, Inject + Matic. Genéva, Switzerland) and stored at 17 °C in ND96 buffer (96 mM NaCl, 2 mM KCl, 1.8 mM CaCl_2_, 1 mM MgCl_2_, 5 mM sodium pyruvate, 5 mM HEPES, pH 7.6) as described previously^40^. P2X1R mutants were available from previous studies^21^,^41^. Media was changed daily prior to recording 3–7 days later. All animal experiments were done with the approval by and accordance with the relevant regulatory guidelines and standards set by the University of Leicester.

### Electrophysiological recordings

Two-electrode voltage clamp recordings (at a holding potential –60 mV) were performed on cRNA-injected oocytes using a GeneClamp 500B amplifier with a Digidata 1322 analog-to-digital convertor and pClamp 8.2 acquisition software (Axon Instruments, Molecular Devices, Foster City, CA, USA), as described previously^40^.

### Voltage-Clamp Fluorometry

Voltage-clamp fluorometry recordings were undertaken as previously reported^21^. Two-electrode voltage clamp recordings were conducted on cRNA-injected oocytes bathed in ND96 using an Axoclamp 900A amplifier with a Digidata 1440A analog-todigital converter and pClamp 10.2 acquisition software (Molecular Devices). VCF recordings were performed using a custom organ bath, designed to apply drug solutions directly to the underside of the oocyte^42^. To ensure rapid solution exchange, drugs were applied using a ValveLink 8 perfusion system (AutoMate Scientific, Berkeley, CA) electronically controlled by the pClamp protocol. Oocytes were imaged using a Nikon Diaphot 200–inverted microscope equipped with HQ545/30 exciter, Q565LP dichroic and HQ572LP emitter filter set, with OptoLED lite light source (Cairn Research, Faversham, UK). Fluorescence was detected by a photomultiplier tube (Cairn Research) installed to the side port of the microscope, with data recorded using pClamp 10.2. Oocytes were incubated in 5 μM MTS-TAMRA (thiol reactive to label available cysteine residues, Toronto Research Chemicals, Toronto, Canada) for 60 seconds, washed in ND96 and stored on ice in the dark before VCF recordings. During VCF recordings, the baseline fluorescence output of the oocyte was recorded for 10 seconds before drug application. Any detected change in fluorescence was calculated as the percentage change from the preapplication level. The fluorescence recovery from receptor desensitization was analyzed by normalizing the fluorescence output during agonist washout to a time point directly after the end of the ATP application (termed -100% from baseline), with any difference calculated as a percent of change (for clarity only, the data points collected each second are shown). In control studies, measuring fluorescence in a plate reader, agonists ATP, Ap5A and αβmeATP (up to 10 mM) had no effect on MTS-TAMRA fluorescence showing that they do not directly interact with the fluorophore, therefore any changes seen in the VCF studies results from a change in the environment of the MTS-TAMRA and not due to direct quenching or interaction with the agonist. However due to cost we did not test the deoxy forms of ATP, but as the variation from ATP is only removal of a hydroxyl group it seemed unlikely that this would lead to direct quenching.

### Data Analysis

Individual normalized concentration response curves were fitted with the Hill equation (variable slope) with GraphPad Prism 6. To characterize any difference in the time course of the current and fluorescence changes the percentage of maximal change in fluorescence (Fmax) at peak current (Imax) was determined (%Fmax at Imax). The amplitude and time to peak current and fluorescence changes were measured using Clampfit 10.2 (Molecular Devices). Once Imax was reached, the fluorescence change at Imax was measured at that time point and calculated as a percentage of Fmax. Data collected from electrophysiological and VCF studies are shown as mean ± S.E.M. Any differences between the means were determined by either Student’s t test or one-way analysis of variance with the Bonferroni post-test as appropriate. Unless stated, n ≥ 3 for all average data.

## Additional Information

**How to cite this article**: Fryatt, A. G. *et al*. Mechanistic insights from resolving ligand-dependent kinetics of conformational changes at ATP-gated P2X1R ion channels. *Sci. Rep*. **6**, 32918; doi: 10.1038/srep32918 (2016).

## Supplementary Material

Supplementary Information

## Figures and Tables

**Table 1:** H-bond frequencies for ATP in P2X1 K68A-mutant MD simulations, and for ATP and derivatives in P2X1 wild type MD simulations.

	K68		K70	N290	R292	K309		T186
	OG	OA	OG	OB	OG	OG	OB	N1
*ATP (K68A)	93.0 ± 4.8	98.5 ± 5.8	89.8 ± 2.5	55.4 ± 3.6	88.4 ± 4.6	87.1 ± 3.2	82.0 ± 3.5	59.4 ± 3.6
**ATP (K68A)	n.a.	n.a.	63.5 ± 0.7	20.1 ± 2.2	79.8 ± 4.6	84.2 ± 2.6	62.3 ± 5.1	36.9 ± 3.1
***ATP (K68A)	n.a.	n.a.	35.6 ± 1.7	0.0 ± 0.0	57.4 ± 3.8	79.6 ± 2.9	45.5 ± 4.0	23.5 ± 0.1
ATP	93.8 ± 3.1	98.7 ± 3.9	93.4 ± 1.5	68.4 ± 0.3	88.6 ± 3.1	92.8 ± 2.3	85.5 ± 2.4	59.6 ± 1.7
3′-deoxy ATP	94.1 ± 3.6	98.7 ± 4.2	92.8 ± 1.3	61.1 ± 4.4	75.3 ± 3.1	85.0 ± 2.1	86.0 ± 2.5	57.0 ± 2.5
2′-deoxy ATP	92.5 ± 1.8	96.0 ± 2.8	81.2 ± 1.0	50.0 ± 4.8	83.7 ± 2.0	85.7 ± 1.2	77.8 ± 2.1	55.3 ± 1.8

H-bond present in % of frames (extracted in 10 ps time steps) for ATP, 3′-deoxy ATP and 2′-deoxy ATP. Mean and stdev are derived from six replicates from two trajectories (ATP, 3-deoxy ATP and 2′-deoxy ATP), six replicates from three trajectories (*ATP) and three replicates from three trajectories (**ATP and ***ATP). H-bonds were defined by the cpptraj command hbond which validates trajectories based on a library of distance and angle criteria. Columns OA, OB and OG refer to hydrogen bonds to α, β and γ-phosphate oxygen atoms, N1 to the adenine ring.

^*^Wild-type sites in K68A simulations; **K68A sites all frames; ***K68 sites only frames where distance Pγ-C2′ > = 5.0 Å.
