# Supplementary material for: Mechanistic insights from resolving ligand-dependent kinetics of conformational changes at ATP-gated P2X1R ion channels
Source: Sci Rep. 2016 Sep 12;6:32918. doi: 10.1038/srep32918 (PMC5018734; doi:10.1038/srep32918)
Supplement: Supplementary Information [file srep32918-s1.pdf]

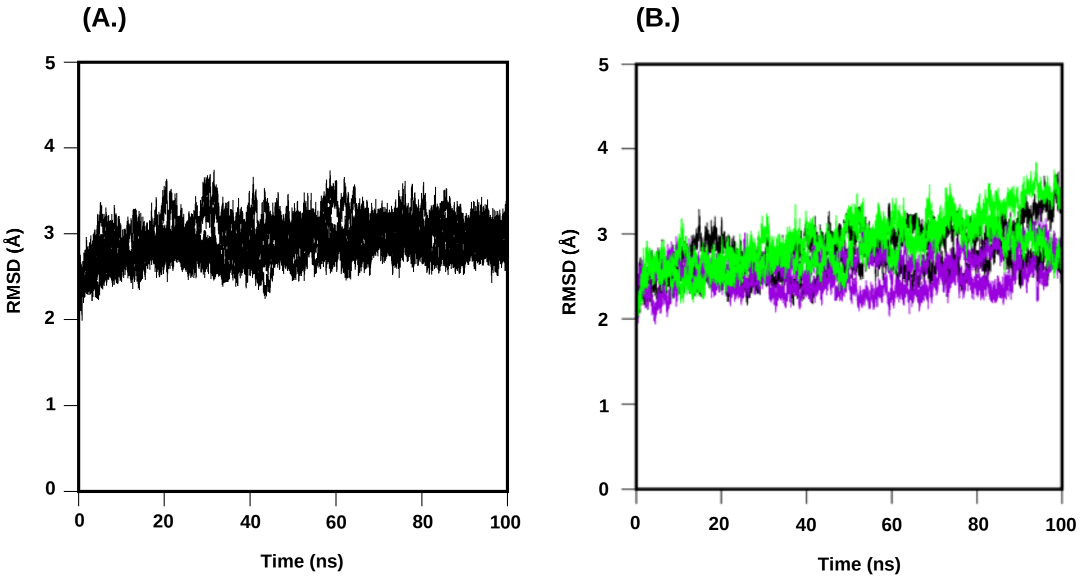

Supplemental Figure 1. Molecular dynamics simulations. **A)** Cα-rmsd plots for simulations of the hP2X1 receptor with one subunit bearing a K68A mutation. **B)** Cα-rmsd plots are shown for hP2X1 simulations with ATP (black), 2- deoxy (green) and 3-deoxy (violet) bound to the receptor.

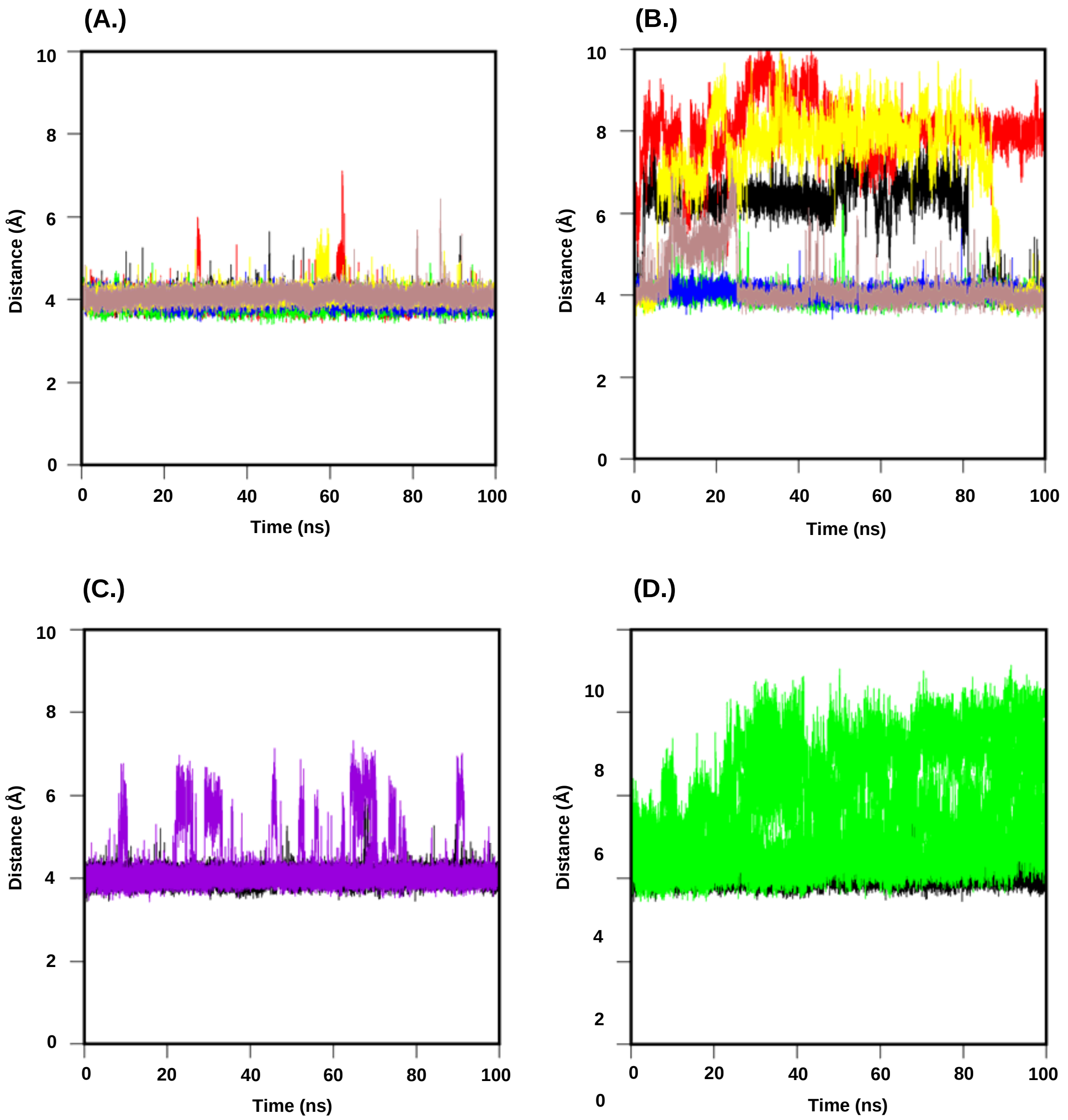

Supplemental Figure 2. Molecular dynamics simulations – ATP conformation monitored by γP – C2'distances. A,B) hP2X1 simulations with ATP bound showing native sites (A) and K68A mutated sites (B). Replicate simulations are distinguished by colour. **C,D)** hP2X1 simulations with ATP (black), 2- deoxy (green) and 3-deoxy (violet) bound to the receptor.
